# Gut barrier structure, mucosal immunity and intestinal microbiota in the pathogenesis and treatment of HIV infection

**DOI:** 10.1186/s12981-016-0103-1

**Published:** 2016-04-11

**Authors:** Camilla Tincati, Daniel C. Douek, Giulia Marchetti

**Affiliations:** Clinic of Infectious, Diseases Department of Health Sciences, San Paolo Hospital, University of Milan, Via di Rudini’ 8, 20142 Milan, Italy; Human Immunology Section, Vaccine Research Center, National Institute of Allergy and Infectious Disease, National Institutes of Health, Bethesda, MD USA

**Keywords:** Microbial translocation, Gastrointestinal barrier, Mucosal immunity, Microbiota

## Abstract

Over the past 10 years, extensive work has been carried out in the field of microbial translocation in HIV infection, ranging from studies on its clinical significance to investigations on its pathogenic features. In the present work, we review the most recent findings on this phenomenon, focusing on the predictive role of microbial translocation in HIV-related morbidity and mortality, the mechanisms by which it arises and potential therapeutic approaches. From a clinical perspective, current work has shown that markers of microbial translocation may be useful in predicting clinical events in untreated HIV infection, while conflicting data exist on their role in cART-experienced subjects, possibly due to the inclusion of extremely varied patient populations in cohort studies. Results from studies addressing the pathogenesis of microbial translocation have improved our knowledge of the damage of the gastrointestinal epithelial barrier occurring in HIV infection. However, the extent to which mucosal impairment translates directly to increased gastrointestinal permeability remains an open issue. In this respect, novel work has established a role for IL-17 and IL-22-secreting T cell populations in limiting microbial translocation and systemic T-cell activation/inflammation, thus representing a possible target of immune-therapeutic interventions shown to be promising in the animal model. Further, recent reports have not only confirmed the presence of a dysbiotic intestinal community in the course of HIV infection but have also shown that it may be linked to mucosal damage, microbial translocation and peripheral immune activation. Importantly, technical advances have also shed light on the metabolic activity of gut microbes, highlighting the need for novel therapeutic approaches to correct the function, as well as the composition, of the gastrointestinal microbiota.

## Background

A decade ago, microbial translocation was described as an underlying cause of T-cell activation in HIV infection. It was shown to decrease in the course of combination antiretroviral therapy (cART) but to persist at higher levels compared to HIV-uninfected controls.

In recent years, studies have investigated the role of microbial translocation markers in predicting the outcome of HIV infection and have also shed new light on the interplay of pathogenic features of microbial translocation, in particular, gut barrier structure, mucosal immune function, composition and metabolic activity of the intestinal microbiota. Further, research studies have also demonstrated the limited effect of cART in antagonizing microbial translocation and the mechanisms by which it arises, thus prompting the investigation of novel interventional strategies.

The aim of the present review is to examine the most recent findings on microbial translocation, focusing on its clinical significance, pathogenic features and therapeutic approaches.

### Microbial translocation and clinical outcome in HIV infection

T-cell activation is a hallmark of HIV infection and associates with clinical progression in untreated disease [1–3]. Following the discovery of microbial translocation as a mechanism for immune activation [4–6], different groups assessed the role of circulating bacterial components in predicting the outcome of HIV infection. In this respect, plasma levels of sCD14 and lipopolysaccharide (LPS) were first described as independent predictors of mortality [7] and disease progression [8] in chronically-infected individuals; similar results were recently found in the setting of spontaneous control of HIV viremia [9] (Table 1). Conflicting data exist, however, on the role of microbial translocation in predicting clinical events in the course of treated infection. One study demonstrated that measures of LPS-induced innate immune activation (sCD14), gut epithelial barrier dysfunction (I-FABP; zonulin-1), coagulation (D-dimer) and inflammation (hs CRP, sTNFRI) were able to predict mortality in individuals on cART [10] (Table 1). In accordance with these findings, also high levels of similar markers prior to the initiation of antiretrovirals were shown to predict non-AIDS morbid events on stable treatment [11] (Table 1). In contrast however, administration of cART in elite controllers did not affect markers of microbial translocation and inflammation [12]. In a recent report, our group showed that pre-cART levels of CRP, but not sCD14, LPS or EndoCAb predicted clinical events in a large cohort of treated HIV-infected subjects [13] and suggest, together with other literature evidence, that the occurrence of non-AIDS conditions may be only in part related to gut damage and microbial translocation (Table 1).

**Table 1:** **Markers of disease progression and their biological antagonists in HIV infection**

Marker	Antagonist	Untreated HIV infection		Treated HIV infection	
		Clinical significance	Evidence of clinical benefit following intervention	Clinical significance	Evidence of clinical benefit following intervention
LPS	Sevalamer Rifaximin	Predictor of disease progression [8]	No significant change in naïve subjects following sevelamer [72]	No association with clinical events [7, 13]	No significant change in subjects with CD4+ <350/mmc following rifaximin [74]
sCD14	Sevelamer Mesalamine Rifaximin	Predictor of disease progression in HIV controllers [9]	No significant change in naïve subjects following sevelamer [72]	Predictor of mortality [7], also in subjects with a history of AIDS [10] No association with clinical events [13]	No significant change in subjects with CD4+ <350/mmc following mesalamine [73] and rifaximin [74]
EndoCAb	Sevelamer Rifaximin	Predictor of disease progression in HIV controllers [9]	Unknown	No association with clinical events [7, 13]	Unknown
I-FABP	Not applicable	Unknown	Unknown	No association with clinical events [7] Predictor of mortality in subjects with a history of AIDS [10]	Unknown
Zonulin	Not applicable	Unknown	Unknown	Predictor of mortality in subjects with a history of AIDS [10]	Unknown
D-dimer	Mesalamine	Unknown	Unknown	Predictor of all-cause mortality in subjects on intermittent therapy [75] Predictor of mortality in subjects with a history of AIDS [10] Predictor of non-AIDS morbid events both prior to treatment initiation and at year 1 [11]	No significant change in subjects with CD4+ <350/mmc following mesalamine [73]
hs CRP	Mesalamine	Unknown	Unknown	Predictor of mortality in subjects with a history of AIDS [10] Predictor of AIDS and non-AIDS events prior to treatment initiation [13]	Unknown
sTNFRI	Mesalamine	Unknown	Unknown	Predictor of mortality in subjects with a history of AIDS [10] Predictor of non-AIDS morbid events both prior to treatment initiation and at year 1 [13]	Unknown
KTR	IDO1-inhibiting bacteria? [64]	Unknown	Unknown	Predictor of mortality in subjects with a history of AIDS [10] Predictor of non-AIDS morbid events both prior to treatment initiation and at year 1 [13] Predictor of mortality both prior to treatment and at month 6 [47] Predictor of unsuccessful immune recovery at month 12 [47]	No significant change in subjects with CD4+ <350/mmc following mesalamine [73]
IL-6	Mesalamine Chloroquine	Unknown	Contrasting results following chloroquine administration [76]	Predictor of all-cause mortality in subjects on intermittent therapy [75] Predictor of non-AIDS morbid events both prior to treatment initiation and at year 1 [11]	No significant change in subjects with CD4+ <350/mmc following neither mesalamine [73] nor chloroquine [76]

*LPS* lipolysaccharide, *sCD14* soluble CD14, *EndoCAb* endotoxin core antibodies, *I*-*FABP* intestinal fatty acid binding protein, *hs CRP* high sensitivity C reactive protein, *sTNFRI* soluble tumor necrosis factor receptor I, *AIDS* acquired immune deficiency syndrome *KTR* K ynurenine tryptophan ratio, *IDO1* indoleamine 2,3-dioxygenase 1

Collectively, studies have shown that microbial translocation markers may predict clinical outcome in the setting of untreated HIV infection but may not be as valuable in cART-experienced individuals. It must be pointed out, however, that the majority of reports on treated subjects were conducted in extremely diversified and antiquated patient populations. In order to fully grasp the clinical utility of these parameters, future investigations will need to be designed for contemporary study populations, i.e. subjects starting therapy with high CD4+ T-cell counts and no history of AIDS complications.

### Pathogenic features of microbial translocation in HIV infection

#### Structure of the gastrointestinal epithelial barrier

Impairment of the gut barrier is a prerequisite for microbial translocation and subsequent immune activation. Indeed, there is evidence of HIV-related damage to the epithelial barrier [14] as well as the presence of bacteria and microbial components in the *lamina propria* of untreated SIV-infected macaques [5, 15] and HIV-infected individuals [16]. In accordance with these findings, a recent study demonstrated a strong association between circulating markers of intestinal damage and measures of LPS-dependent immune activation, but not HIV RNA load, in a large cohort of HIV-infected cART-naive subjects [17], suggesting that microbial translocation and immune activation may be independent of viral replication in the chronic phase of untreated infection. Further, these results are consistent with the high levels of (microbial-induced) immune activation observed in some individuals during fully suppressive therapy [4, 18–20] and anticipate the question of whether cART, as well as its timing and duration, is able to revert the damage of the gastrointestinal tract.

**Fig. 1:**
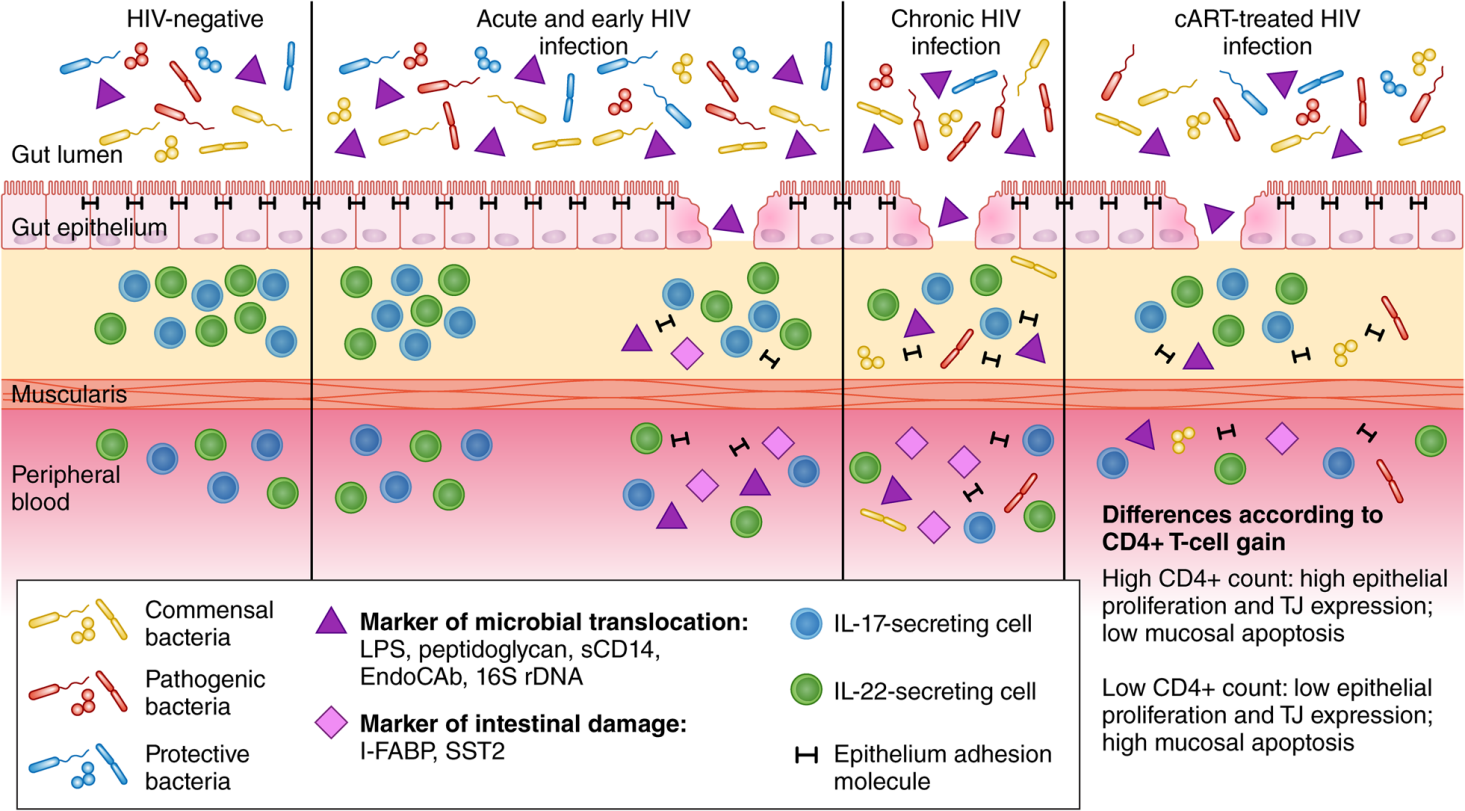
The *continuum* of gastrointestinal abnormalities in treated and untreated HIV disease. In HIV-negative individuals, a highly diversified microbiota resides in the gut lumen and is shielded from the lamina propria, thus preventing the translocation of bacteria and microbial products to the peripheral blood. Microbial translocation is blocked thanks to the structural integrity of the epithelial barrier and function of gut-resident immune cells, e.g. IL-17- and IL-22- producing cells. In the acute phase of HIV disease, no significant alterations of luminal bacteria and epithelial barrier have been reported. Indeed, markers of microbial translocation (LPS, peptidoglycan, EndoCAb, sCD14, 16S rDNA) and enterocyte damage (I-FABP, sST2) were not detected in plasma samples of acutely-infected individuals and both mucosal and peripheral Th17 cells were only slightly decreased in the earliest phases of HIV infection. Following 6 months from infection (early HIV infection), however, a rise in parameters of microbial translocation and gut impairment was measured in peripheral blood, suggesting initial disruption of mucosal integrity. In the chronic stages of untreated HIV disease, new research studies have demonstrated profound alterations of the intestinal epithelium, with marked impairment of proteins forming the gut junctional complex (JC; cadherin, claudins, zonula occludens 1) which can be found in the peripheral blood. These anatomical alterations are accompanied by changes in the composition and function of the gut microbiota as well as the reduction of IL-17- and IL-22-secreting cell numbers, allowing for the translocation of pathogenic bacteria. Combination antiretroviral therapy (cART) is able to correct gut abnormalities only in part. Persistent defects in JC protein expression, microbial composition and immune function have in fact been described in aviremic individuals. Importantly, these defects appear to be differentially expressed according to the degree of CD4+ T-cell recovery on cART

Two studies addressed the effect of early treatment administration in preserving the structure and function of the gut epithelial barrier. An important contribution in the field was made by a study on HIV-infected subjects enrolled in the acute phase of disease at a median time-point of 42 days from infection. At the time of enrolment, subjects displayed comparable I-FABP levels to those measured in uninfected controls, as well as undetectable peptidoglycan and bacterial 16S rDNA. Following 6 months from enrolment, the authors found a significant increase in intestinal damage, yet not microbial translocation parameters, in all study participants regardless of treatment receipt [21]. The findings of this report not only shed light on when structural damage and microbial translocation occur in the course of HIV infection, i.e. the former arises approximately 6 months from infection and the latter in tardier stages of disease as previously suggested [6] (Fig. 1), but also entail that immune activation may not be driven uniquely by microbial translocation in acute HIV infection. Further, these results also show that the initiation of cART in this setting may not suffice to halt the profound alterations that occur within the gastrointestinal tract. Consistent with these findings, a study on individuals for whom HIV acquisition was estimated less than 180 days earlier demonstrated that timely cART administration did not improve plasma levels of gut mucosal dysfunction markers (I-FABP/sST2), thus confirming the persistence of gut barrier impairment in this setting [22].

Cross-sectional studies have also shown the presence of gut mucosal injury and microbial translocation in long-term cART-treated individuals. In an earlier study, elevated plasma levels of E-cadherin, an epithelial adhesion molecule, were found in aviremic individuals, suggesting the disruption of epithelial barrier integrity [23] (Fig. 1). More recently, the expression of tight junction (TJ) complex proteins and genes was shown to progressively decrease in a proximal-to-distal manner within the colonic epithelium of virologically-suppressed subjects who had started cART with a median CD4+ T-cell count of <200/mmc, yet presented favourable immune competency at the time of the study (CD4 T-cell count >500/mmc) [24] (Fig. 1). Similarly, decreased expression of CLDN4 was found in the colon in aviremic individuals with a variable degree of immune recovery on cART [25], posing the question as to whether differences in TJ complex protein expression exist according to the extent of CD4+ T-cell gain, given literature reports of higher T-cell activation and microbial translocation in treated subjects with persistent CD4+ T-cell deficiency [4, 6, 18–20].

In this respect, our group conducted a comparative analysis of the gut junctional complex (JC) in subjects with divergent immune responses to virologically-suppressive treatment (Immunologic Non Responders, INR: CD4+ T-cell count <350/mmc; Full Responders: CD4+ T-cell count >350/mmc) and showed that the expression of JC proteins in colonic tissue was significantly lower in HIV-infected subjects with incomplete CD4+ T-cell restoration [26] (Fig. 1). Our findings complement an earlier observation which demonstrated low Ki67 indexes in colorectal biopsies of INR and an inverse correlation between epithelial proliferation and mucosal apoptosis, thus suggesting that the latter plays a key role in the loss of epithelial integrity in HIV-infected individuals with poor immune-recovery on cART [27] (Fig. 1). Taken together, these reports point to the persistence of structural damage of the colon in treated individuals and suggest decreased gene and protein expression as well as increased cellular death as underlying mechanisms of gut injury in this setting.

Contrasting results, however, have been produced in terms of whether structural impairment of the gastrointestinal barrier actually translates into increased mucosal permeability and translocation of microbial products in the course of aviremic HIV disease. On the one hand, the finding of a correlation between decreased JC proteins, elevated plasma LPS [24], heightened mucosal apoptosis [27] and, possibly, systemic immune inflammation [24] allows for the speculation that gut impairment causes microbial translocation. On the other, the lack of an association between the expression of mucosal TJ genes and systemic measures of gut leakiness/damage [25], together with our results of comparable microbial translocation in cART-treated individuals regardless of the marked differences in gut barrier structure [26], suggests the interplay of other factors in the containment of such phenomena.

Despite solid evidence for profound damage to the gastrointestinal tract in long-term treated subjects, further studies need to be carried out to clarify whether mucosal loss truly accounts for decreased functionality of the epithelial barrier. Further, with the exception of results from the Thai cohort discussed below [28], data are lacking in the setting of acute/early HIV infection with findings restricted to circulating markers of gut damage and few results on gene and protein expression of the JC. In this respect, longitudinal sampling of the gut mucosa in acutely-infected individuals will shed light on when gastrointestinal impairment precisely arises and whether it may be reverted by cART.

#### Mucosal immune function

A crucial question when assessing mucosal immunity in the course of HIV infection is the fate of gut CD4+ T-cells. Many studies have shown that mucosal CD4+ T-cells expressing CCR5 as well as peripheral HIV-specific CD4+ lymphocytes expressing the gut-homing markers integrin β7 and CCR6 are depleted in both acute and chronic phases of HIV/SIV infection [29–33] and partially restored by cART [34]. In this context, a recent study demonstrated that initiation of treatment in the acute phases of infection may be able to preserve gut CD4+ T-cell numbers, however, administration of therapy in chronic HIV disease not only fails to restore the frequencies of these cells, but also has a limited effect on their differentiation [35].

Aside from CD4+ T-cells, the gastrointestinal tract is home to other populations known to guard immune homeostasis and health at mucosal sites, specifically IL-17- and IL-22-producing cells [36, 37] which were first postulated to have a role in the mucosal-associated pathogenesis of SIV [38].

A limited number of reports conducted in the course of acute HIV infection convene that peripheral and mucosal Th17 populations are minimally affected in the earliest phases of disease [21, 28] (Fig. 1). The frequency of circulating Th17 cells was shown to remain stable over the first 6 months of disease (Fig. 1) and did not vary following treatment administration in this setting [21]. In contrast, mucosal Th17 subsets appeared to decline approximately 3 weeks from infection [28] (Fig. 1); initiation of antiretroviral treatment in Feibig I/II stages was shown to prevent their numerical and functional loss, while initiation in later stages was able to correct only Th17 frequency but not polyfunctionality [28].

Studies confirm the depletion of Th17 and Th22 populations in chronic HIV infection in peripheral blood [39] and mucosal sites [40], yet counterarguments exist regarding reconstitution of these subsets in the course of antiretroviral treatment (Fig. 1). Indeed, while persistent impairment of the Th22/Treg ratio has been described in the peripheral blood of individuals on therapy [39], IL-22-secreting populations in the gut appear to be efficiently restored regardless of when cART is initiated [40]. Similarly, while cART appears to rapidly normalize the frequencies of mucosal Th17 cells [41, 42], it may take longer to restore their capacity to secrete IL-17 [40] and other cytokines (IL-22, IFN-γ, TNF) [42]. Of note, building on prior evidence of the Th17 lineage marker CCR6 as an indicator of memory cells imprinted with a transcriptional program favorable to HIV replication [43], a novel report recently put forward permissiveness to abortive and/or integrative infection of naïve-like Th17 precursors as a possible mechanism underlying Th17 deficiency in cART-treated subjects [44].

Studies have also assessed the role of these cells in the pathogenesis and outcome of HIV infection. Disruption of the Th17/Treg balance in peripheral blood and rectosigmoid tissue was initially found to feature progressive HIV disease and associate with the induction of indoleamine 2,3-dioxygenase 1 (IDO1) [45]. These findings were later confirmed by a study on naïve subjects which showed that the frequency of Th17 and Th22 subsets as well as their ratio to Treg cells appeared to negatively correlate with CD8+ T-cell activation, microbial translocation and IDO1 activity, further suggesting their contribution to systemic immune activation and mucosal deficiency in HIV [39]. Indeed, immune-regulatory skewing of mucosal Th17 cell function characterized by an increased IL-10/TNF-α ratio was found to be an independent predictor of immune activation in untreated disease [42]. Importantly, these defects were shown to persist in the course of antiretroviral treatment, given the inverse correlation between IL-17-producing Mucosal Associated Invariant T (MAIT) cells and IDO1 activity in cART-treated individuals, especially in those presenting poor CD4+ recovery [46]. These results strengthen the hypothesis that IDO1-dependent immune deregulation may influence clinical outcome [47] and challenge CD4+ reconstitution on cART [47, 48].

In the past few years, research on mucosal immunology has evolved from the investigation of CD4+ T-cell depletion/reconstitution to the study of the homeostasis of other subsets, which may affect the structure and function of the gastrointestinal tract. Given the difficulty in examining gut tissue due to recovery/sampling issues in HIV-infected humans, the majority of studies have focused on a limited number of mucosal cell subsets. Future research should address the complex interactions between multiple cell populations at mucosal surfaces and further understand their role in the pathogenesis of microbial translocation, immune activation and inflammation in HIV infection.

#### Composition and metabolic activity of the intestinal microbiota

The intestinal microbiota is essential to the proper function and development of the host and plays a key modulatory role in many disorders [49].

In HIV infection, disruption of the physiological gut microbiota occurs early in the course of disease [50] and may be linked to immune imbalances in the gastrointestinal tract [51] and peripheral blood [51, 52]. Recent studies have shed new light on the contribution of dysbiosis to mucosal and systemic dysfunction in both progressive and treated HIV infection.

cART-naïve subjects have been shown to display a dysbiotic recto-sigmoid-adherent community, enriched in Proteobacteria and depleted in Bacteroidia, which associates with heightened T-cell activation in the blood and gut, lower mucosal IL-17/IL-22 secretion, higher plasma Kynurenine Tryptophan Ratio (KTR, index of IDO1 activity), and inflammatory markers [53]. These findings have been confirmed by a more recent report, which showed abundances of colonic Proteobacteria and decreased Firmicutes in HIV-infected, untreated individuals, as well as an association between microbial impairment and activated mucosal T and myeloid dendritic cells [54] (Fig. 1).

Alterations in the composition of the intestinal microbiota do not seem to be reversed by cART. In one study, cART-treated subjects showed decreased microbial diversity in the right colon and terminal ileum compared to uninfected controls, with the loss of commensal as well as a gain of some pathogenic bacterial taxa [55] (Fig. 1). In another report, the presence of Bacteroidetes in the colon of aviremic individuals correlated with expression of inflammatory genes in the same district (IFNG, IL1B) [25], implying that intestinal dysbiosis may contribute to altered mucosal gene expression. Furthermore, profiling of the fecal microbiota showed relative abundance of certain Proteobacteria and depletion of Bacteroidetes, changes which associated with markers of microbial translocation and systemic inflammation [56]. Interestingly, a longitudinal study on men starting cART early in the course of HIV infection demonstrated a beneficial effect of fecal Lactobacillales in modulating the immune function during infection, given their association with higher CD4+ T-cell count in the blood and gut, less microbial translocation, less systemic immune activation and gut T-cell proliferation [57]. Taken together, these studies suggest that a fine regulation of the gastrointestinal microbiota may influence disease outcome in treated HIV-infected subjects, possibly though decreases in mucosal and systemic inflammation.

In this respect, recent technical advances have shed light not only on the composition of resident bacteria but also on the functional activity of the microbiota, which may actually be more critical to human health than the identity of the species providing it [49].

In SIV-infected non-human primates, increased metabolic activity of Proteobacterial species was found within the colonic lumen [58]. In HIV-infected subjects, enrichment of gut resident bacteria capable of metabolizing tryptophan catabolism was found [53]. In keeping with this observation, imputed metagenomic functions of the rectal microbiota, including amino acid metabolism and vitamin and sidephore biosynthesis, showed significant differences between healthy controls and cART-naïve subjects [59]. Most recently, a fine characterization of the functional gene content of the gut microbiota and its metabolic pathways in cART-treated subjects showed enrichment of genes involved in various pathogenic processes, LPS biosynthesis, bacterial translocation and other inflammatory pathways as well as significant interactions between the bacterial community, their altered metabolic pathways and systemic markers of immune dysfunction [60].

Collectively, these findings are consistent with the profound changes affecting the composition of the gut microbiota both in untreated and treated HIV infection. Developments in the fields of metagenomics and metabolomics have also shown skewed functions of intestinal bacteria in HIV-infected individuals and may aid in the elaboration of interventional strategies for the treatment of HIV.

### Approaches to reverse mucosal immune impairment, intestinal dysbiosis and microbial translocation

Given the lack of therapeutic interventions to specifically target the restoration of the epithelial barrier structure, work has been carried out in the attempt to reverse mucosal immunity abnormalities, microbial dysbiosis and translocation.

Restoration of the intestinal microbiota was first approached through the administration of prebiotics to HIV-infected individuals [61, 62]. Data in SIV-infected macaques have shown that co-administration of antiretrovirals and prebiotics resulted in increased frequency and functionality of gut antigen-presenting cells, enhanced reconstitution and functionality of CD4+ T-cells as well as reduced fibrosis of lymphoid follicles in the colon [63]. In a recent report, chronically-infected macaques supplemented with a Lactobacillus-containing probiotic exhibited decreased IDO1 activity, providing support for the role of these species in modulating mucosal immune homeostasis through the inhibition of an immunomodulatory enzyme [64] (Table 1). In HIV-infected humans, partially contrasting results stemmed from new reports on the use of probiotics in cART-treated subjects: on the one hand, decreases in microbial translocation and inflammation markers as well as T-cell activation were demonstrated in individuals randomized to receive oral supplementation with probiotics [65, 66]; on the other, administration of similar components to HIV-infected individuals with CD4+ T-cell count <500/mmc resulted in the improvement of the microbiota and decreases in D-dimer levels, yet had no effect on microbial translocation or T-cell activation [67].

Experimental strategies aimed at correcting mucosal immune imbalances have been carried out in humans and animal models. In particular, recombinant human IL-7 was shown to improve rectosigmoid abnormalities in individuals with incomplete CD4+ T-cell reconstitution on cART through increases in mucosal CD4+ T-cell counts and decreases in neutrophil infiltration [68]. The administration of IL-21 to SIV-infected macaques lead to the amelioration of total and SIV-specific T-cell function and higher levels of intestinal Th17 cells which associated with reduced intestinal proliferation, microbial translocation and systemic activation/inflammation [69]. In another study, a combination strategy consisting of the co-administration of IL-21 and probiotics in treated SIV-infected animals triggered the expansion of polyfunctional Th17 cells and limited the translocation of pathobionts [70]. A recent report expanded the boundaries of existing knowledge in the field of IL-21 immunotherapy in combination with cART by demonstrating that this strategy is able to restore intestinal Th17 and Th22 cells and reduce mucosal as well as peripheral immune activation. Of note, IL-21 also accounted for substantial decreases in the viral reservoir, which lasted over time following cART interruption [71].

Finally, approaches to precisely target the passage of microbes from the gut lumen to the systemic circulation have also been attempted. In the macaque model, one study demonstrated that sevelamer, an LPS-antagonizing compound, was able to reduce immune activation and coagulation markers and to constrain viral replication in acutely-infected macaques [15]. In contrast, administration of the same drug to chronically-infected, cART-naïve subjects did not lower microbial translocation, inflammation or T-cell activation parameters [72] (Table 1), pointing to the need of broader interventions able to contrast multiple features of HIV pathogenesis. Similarly, mesalamine and rifaximin, respectively an anti-inflammatory agent and a non-absorbable antibiotic, administered to subjects with poor CD4+ T-cell gain on virologically-suppressive cART, had no effect on markers of peripheral inflammation [73], microbial translocation and CD8+ T-cell activation [74] (Table 1). Possible explanations for these results may be lack of specificity in the former and only partial modification of the microbial community (dysbiosis rather than abiosis) in the latter.

Overall, therapeutic interventions aiming to restore the composition of the intestinal microbiota and limit microbial translocation have been proven to be largely ineffective in the setting of chronic HIV infection for understandable reasons, which in fact highlight the pathogenic complexity of microbial translocation; i.e. failure to target the gastrointestinal tract vis-à-vis epithelial structure, mucosal immunity and microbial composition. Promising data seem to result from immunotherapy strategies tested in the macaque model and may represent a starting point for clinical trials in humans.

## Conclusions

Since its discovery approximately a decade ago as a cause of T-cell activation in HIV infection, microbial translocation has been investigated as a mechanism underlying increased morbidity and mortality in this setting. Studies in untreated cohorts have suggested that microbial translocation parameters may be useful in predicting clinical events, while reports in cART-experienced individuals are less consistent on this matter. A reason for this may be the inclusion of patient populations who started therapy in advanced stage of disease and/or had a history of AIDS-defining events. In this respect, future research on the predictive role of microbial translocation in treated HIV infection need to be carried out in contemporary study populations and define clinical end-points accordingly.

From a pathogenic perspective, extensive work has been carried out on the understanding of the structural and functional impairment of the gastrointestinal tract. While literature reports seem to agree on the HIV-related damage of the gut mucosa, counterarguments exist as to what extent it may translate into increased permeability and translocation of microbial products from the lumen to the systemic circulation, especially in treated disease. In this respect, further research will be required to investigate the interplay between the epithelial barrier, mucosal immune cells and intestinal microbiota. Indeed, recent data have demonstrated a central role of specific cell subsets in antagonizing microbial translocation and systemic T-cell activation/inflammation. Further, thanks to the technical advances in systems biology, studies have shown that impairment of the metabolic functions of the microbiota, rather than its composition, may influence gut and systemic health. Finally, given the divergent reports on the administration of probiotics as well as the lack of encouraging results from the treatment with antibiotics and compounds antagonizing microbial translocation, strategies aiming at the correction of mucosal immune imbalances and gut metabolome may represent promising approaches for the treatment of HIV infection.

## Abbreviations

HIV: human immunodeficiency virus
cART: combination antiretroviral therapy
LPS: lipolysaccharide
sCD14: soluble CD14
I-FABP: intestinal fatty acid binding protein
hs CRP: high sensitivity C reactive protein
sTNFRI: soluble tumor necrosis factor receptor I
AIDS: acquired immune deficiency syndrome
SIV: simian immunodeficiency virus
16S rDNA: 16S ribosomial DNA
sST2: soluble suppression of tumorigenicity 2
TJ: tight junction
CLDN4: claudin 4
JC: junctional complex
INR: immunologic non responder
FR: full responder
IL: interleukin
CCR5 and CCR6: C-C chemokine receptor type 5 and 6
Th: T helper
IFN: interferon
TNF: tumor necrosis factor
Treg: T regulatory
IDO1: indoleamine 2,3-dioxygenase 1
MAIT: mucosal associated invariant T
KTR: kynurenine tryptophan ratio
EndoCAb: endotoxin core antibodies
